# Addressing challenges faced by insecticide spraying for the control of dengue fever in Bangkok, Thailand: a qualitative approach

**DOI:** 10.1093/inthealth/ihy038

**Published:** 2018-06-15

**Authors:** Peeradone Srichan, Saranath Lawpoolsri Niyom, Oranut Pacheun, Sopon Iamsirithawon, Supawat Chatchen, Caroline Jones, Lisa J White, Wirichada Pan-ngum

**Affiliations:** 1 Department of Tropical Hygiene, Faculty of Tropical Medicine, Mahidol University, Bangkok, Thailand; 2 Faculty of Public Health, Thammasat University, Pathumthani, Thailand; 3 Department of Disease Control, Ministry of Public Health, Nontaburi, Thailand; 4 Department of Tropical Pediatrics, Faculty of Tropical Medicine, Mahidol University, Bangkok, Thailand; 5 Nuffield Department of Primary Care Health Sciences, University of Oxford, Oxford, UK; 6 Mahidol-Oxford Tropical Medicine Research Unit, Faculty of Tropical Medicine, Mahidol University, Bangkok, Thailand; 7 Centre for Tropical Medicine, Nuffield Department of Medicine, University of Oxford, Oxford, UK

**Keywords:** Bangkok, Dengue, Qualitative research, Spraying, Surveillance system, Vector control

## Abstract

**Background:**

This study focused on evaluating the fumigation scheme and identifying problems encountered during the operation in the Bangkok Metropolitan Administration area.

**Methods:**

Ten district health officers working in different fumigation teams of the dengue outbreak control programme around Bangkok had participated in an in-depth interview. Five predetermined themes, including (i) dengue surveillance and control strategy, (ii) quality and availability of equipment, (iii) delays, (iv) human resources, and (v) area coverage, and other emerging themes were addressed during the interviews.

**Results:**

Although the staff seemed to know the operation protocol of the dengue surveillance and control programmes well, they encountered some difficulties in accessing households for proper spraying, and a lack of human and material resources, especially during an outbreak. Other emerging themes concerned inefficient communications among the sectors from hospital to district offices, leading to inaccurate or missing patient addresses for spraying, and the lack of community networks and public cooperation for the dengue control programmes.

**Conclusions:**

The findings suggest that coordination among the relevant health sectors to acquire accurate and timely information about dengue cases is essential. Involving community networks should help to improve public engagement with and participation in the surveillance and outbreak control programmes.

## Introduction

Dengue disease has been a major public health issue in Thailand and others tropical countries,^1^ and dengue cases have been reported via the national surveillance system—approximately 100 000 cases per year by the Bureau of Epidemiology, Thai Ministry of Public Health (MoPH).^2^ The transmission pattern of dengue fever in Thailand is seasonal, with the peak during the rainy season (May to October).^3^ However, in the Bangkok Metropolitan Administration (BMA) region, dengue fever tends to circulate throughout the year, with a peak occurring during the rainy season.^2^

**Figure 1.**
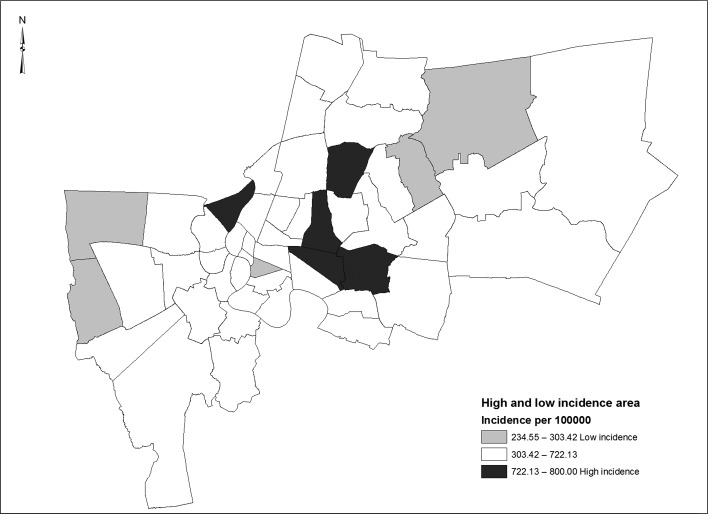
Map showing the 50 districts of Bangkok, with the 5 highest (black) and 5 lowest (grey) dengue incidence districts being sampled.

Bangkok, Thailand’s capital, is divided into 50 geographically defined areas, called districts (Figure 1). Bangkok is a dengue-endemic area, and all four human dengue virus serotypes circulate simultaneously in the city.^4^ Factors thought to influence such a pattern of disease in Bangkok include its population density, infrastructure administration, mosquito density, environmental management, expansion of the city, and changing lifestyles.^5–7^ Heterogeneous spatiotemporal distribution patterns have been observed, as well as a serotype distribution that has varied in both time and place.^8,9^ Only limited success of dengue disease controlling has been achieved by the MoPH.

A fumigation campaign has proven effective for a short-term event such as an outbreak control, but it is often not sustainable.^10^ Integrating several interventions, such as breeding source reduction, awareness of dengue disease and a fumigation campaign, may be required to reach a sustainable goal. Current strategies, such as vector control, will probably be implemented together with a vaccination programme and other interventions.^11^

This study conducted in-depth interviews with district health officers working in district offices around Bangkok, in order to obtain their perceptions on the challenges and successes regarding the fumigation campaigns they were working on directly. Based on this qualitative assessment, the problems encountered during the implementation, which may contribute to the difficulty in controlling dengue in BMA, were identified. Some key components to improve the effectiveness of the fumigation scheme were then suggested and discussed.

### The dengue surveillance reporting system in the Bangkok Metropolitan Authority area

**Figure 2.**
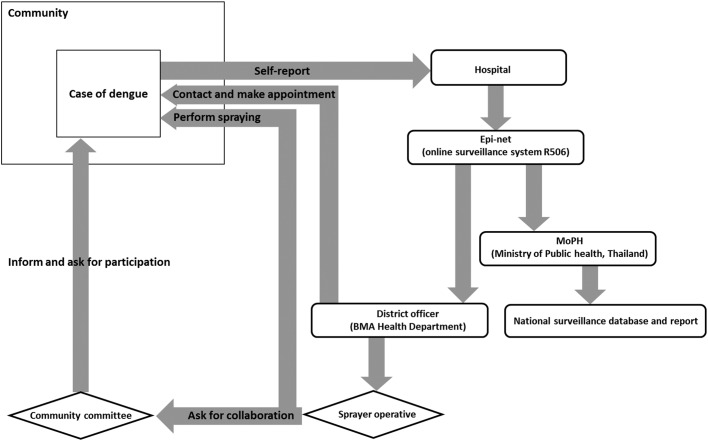
Bangkok’s dengue reporting system. The rounded rectangles represent the reporting process while the diamonds represent the fumigation scheme.

The BMA’s dengue case reporting system operates as follows. Once a suspected dengue case from a community arrives at hospital, i.e. ‘self-report’, usually following 3–4 d of fever or other clinical symptoms, the hospital generally takes 1 d to confirm the case, based on both a laboratory test and on clinical symptoms, according to the WHO guidelines 2012.^12^ When a dengue case is confirmed, the case will be reported in the online surveillance system R506 using an electronic form (Epi-net) to notify the MoPH and the BMA Health Department. The MoPH will then process the data for national surveillance reporting and policy planning. The reporting should occur within approximately 24 h of the dengue confirmation (the rounded rectangles in Figure 2). Upon the notification of the confirmed case, the district officer would form a spraying operative team to contact and make an appointment to visit and spray the patient’s house. The sprayer operatives will access and spray the targeted areas, i.e. the dengue case index house and the houses within a 100 m^2^ radius, within 24 h of receiving the report. This was estimated to cover approximately 50–60 houses per targeted area, depending on the population density of each BMA district.^13^ The sprayer operatives may be assigned to spray the targeted areas either with or without asking the community committees for collaboration; this depends on the district setting (the diamonds in Figure 2). In addition, regarding the MoPH manual of fumigation for dengue prevention and control, the sprayer operatives are advised to outdoor spray all shrubberies and drains within a certain radius where the mosquitoes are likely to rest.^14^

Delays in the system arise due to different health-seeking behaviours, the reporting system itself, and communications between different health sector providers, including hospitals, the district officer and the sprayer operatives, as well as people in targeted areas.

## Materials and methods

### Study districts

The study districts were selected based on the five top and five bottom areas of dengue incidence in 2015 (data from the Urban Prevention and Control of Disease Institute, Department of Disease Control, MoPH) under the hypothesis that the efficiency of the fumigation operation may be different in the two groups. Ten districts were therefore selected to be surveyed (Figure 1), named in Tables 1 and 2 as A, B, C, D, E for the areas of high dengue incidence and F, G, H, I, J for the areas of low dengue incidence.

**Table 1.** Demographics of participants

No.	District	Position	Age	Sex
1.	A	Public health technical officer	28	Female
2.	B	Sanitation technical officer	40	Female
3.	C	Public health technical officer	41	Female
4.	D	Sanitation technical officer	38	Male
5.	E	Public health officer	54	Female
6.	F	Public health officer	47	Male
7.	G	Sprayer operative	36	Male
8.	H	Public health officer	53	Male
9.	I	Public health officer	54	Female
10.	J	Sanitation technical officer	51	Female

**Table 2.** Human and machine resources in all districts

District	Dengue incidence	No. of machines	Age	Machine availability	No. of sprayer operatives	No. of villages
A	High	3	NA	3 available	3	47
B	High	8	>1 y >2 y	4 available 4 sometimes available	3	45
C	High	4	>5 y	4 available	3	17
D	High	6	>5 y	3 available	2	19
E	High	5	NA	3 available	2	24
F	low	7	NA	7 available	16	16
G	low	4	4–6 y	2 available 2 sometimes available	4	64
H	low	3	>2 y	3 available	3	50
I	low	8	>5 y	3 out of service 3 available 2 sometimes available	2	41
J	low	4	>5 y	2 available 2 sometimes available	2	15

### In-depth interview

This study adopted a qualitative approach by conducting face-to-face, in-depth interviews with 10 designated district officers in their local BMA office using some open-ended questions following five predetermined themes. The participants were authorized by the director of each of the selected districts. They were all experts who had experience working in a BMA dengue control programme for at least five years (see supplementary Appendix 1).

### Data analysis

The interviews were audio-recorded and fully transcribed by the first author (PS). Data were read, coded and uncoded repeatedly following the predetermined themes. During this process, initial thoughts, ideas and feelings were also noted. The transcriptions were independently peer reviewed by other experienced researchers, who read the interview transcripts, checked the data analysis and looked at the emerging themes in order to ensure that the information was accurate and there were no missing emerging themes. All analysis was done manually in this study following the guidance of Boyce.^15,16^ The COREQ principle was applied to verify the comprehensive reporting of findings.^17^

## Results

### Demographic description

Ten subjects working under the BMA Health Department participated in the study; all had over 5 y experience of dengue outbreak control activities. There were six female and four male participants. Among the participants, there were four positions:

- two public health technical officers;
- three sanitation technical officers;
- four public health officers;
- one sprayer operative (Table 1).

### Theme I: standard practices of the MoPH and the BMA Health Department

Dengue prevention and control planning was developed by the BMA Health Department, and the strategy was divided into two parts:

- a dengue surveillance plan, for routine public engagement and spraying activities;
- a dengue outbreak control plan, which involved targeted spraying of areas where a dengue case had been notified.

#### Dengue surveillance plan

For the dengue surveillance plan, district health officers usually provided health education regarding the mechanisms of dengue infection and good practices to prevent transmission, reduce severe disease and avoid fatalities. It also involved the promotion of environmental management, e.g. breeding place reduction, big cleaning days (where large-scale cleaning efforts are undertaken by community members), and chemical and biological pest control. These health promotion campaigns often took place in community locations, such as fresh markets, temples and churches.

The interviewees expressed some concerns about finding a suitable place in some settings for the activities outlined in the surveillance plan. For example, in the inner city, where most areas were commercial zones such as shopping malls, places of entertainment and private office buildings, the health staff felt unable to interrupt people on the street to talk about dengue. In addition, gaining access to spray these areas was difficult and perceived as disturbing people in their work environment.

As one participant stated:

We attempt to do campaigns in the city centre, but sometimes we can’t because it would have disturbed the ongoing business activities in those commercial zones.

#### Dengue outbreak control

The second part of the strategy is dengue outbreak control. Fumigation was a routinely used technique for this, which should be performed according to the same standard operating procedure developed between the MoPH and the BMA Health Department. Two commonly used types of spraying machines used in Thailand are fogging machines and ultra-low volume (ULV) liquid machines.

The standard procedure for fumigation included indoor household spraying, where the spraying must be followed by at least 30 min without ventilation, and indoor and outdoor spraying, with a coverage of a 100 m^2^ radius surrounding the patient’s house.

Confirmed cases of dengue fever from hospitals in the BMA area are reported via the online national disease surveillance system, ‘Epi-net’. This is an electronic health record system, which links hospitals, the BMA Health Department and the MoPH.

District health officers retrieve dengue reports from the Epi-net, then print the reports, which include patient information. They will then contact the patient by phone to confirm the dengue case and explain the plan for dengue outbreak control implementation, including the fumigation of their residence and the destruction of mosquito-breeding sources. They will then make an appointment for fumigation and instruct the household members on how to prepare the house. Depending on the district setting, 5 out of the 10 districts contact the head of that community to inform and coordinate with them about the process.

### Theme II: machines—quantity and quality

Two types of spraying machines were used for mosquito control:

- a ULV cold fog generator;
- a SUPERHAWK thermal fog generator.

The preference for these machines varied amongst the community. The ULV cold fog generator was quiet and produced almost invisible droplets, which led some household members to feel as if the staff had not sprayed and/or that there would be no impact of spraying on controlling the vector. On the other hand, the thermal fog generator had a very loud operating noise and produced large amounts of thick smoke, although some people felt reassured by this and expressed more trust that the control would work.

One interviewee said:

Villagers usually preferred fogging to the ULV, because they believed that the smoke could attack mosquitoes better.

Table 2 presents the difference between the proportion of spraying machines and human resources in each of the districts surveyed. The efficiency of spraying represented by the availability of machines and the number of staff in the spraying operative team showed no difference between the districts of high or low dengue incidence. Spraying machines were not available at all times in districts B, G, I and J. In these districts, about half of the machines were frequently not available or out of service.

During the wet season, both staff and spraying machines were overstretched due to the large number of fumigation operations that were necessary. There was no rationale used for determining the appropriate numbers of machines and operatives per district; this varied by budget spent on them at a district level. Furthermore, maintenance service contracts for spraying machines were provided for just 1 y by the suppliers. After this time, when machines broke down they were sent to the company for repair, who took at least 1 mo to repair and return the machines. Often the sprayer operatives would try to fix the machines themselves, although they lacked the proper training and skills to do so. Most of the machines were at least 5 y old.

One participant stated that:

SUPERHAWK machines were usually used. Starting the machines was difficult. It needed special skills and training, which should have been provided by the manufacturer. There was a lack of machine maintenance knowledge among the sprayer operatives. SUPERHAWKs use diesel to run a motor and, when spraying for a long time, the motor might possibly burn out.

Another stated:

We try to fix the spraying machine ourselves, but we do not know how to, and also it is hard to find some spare parts for the machines. When we sent them back to the company, it often took a long time to fix and to be sent back to us.

### Theme III: response times

The district health officer from the BMA Health Department usually checks the Epi-net reporting system twice a day for dengue case reports. On seeing a case report the officer would then start coordinating with the environmental sanitation division at the district level to arrange for fumigation. Ideally, the process from case confirmation until spraying should be completed within 48 h.

Incomplete or missing patient information, such as name, address and contact number, was reported to be a problem. Patients’ addresses were often incorrect or lacked a contact number; thus the time between case finding and fumigation of the patient’s house and surrounding area was delayed. The district health officer would sometimes make an attempt to determine a patient’s address by searching in the civil registration database and collaborating with the local community committee.

One officer said:

Sometimes, we could not contact patients, i.e. patient contact information was inaccurate, or missing a phone number. In some cases, we ended up with no spraying being done.

All interviewees described this problem, and one of them stated:

‘Delayed reporting occurs in some cases, for example we sometimes receive a case notification a week after the patient has already been discharged from hospital.’

The electronic Epi-net system reportedly frequently crashed, rendering it inaccessible and leading to delays in case notification. In some cases, delays in reporting could be at least a week. One reported:

‘We often had problems accessing the Epi-net system, due to either the software itself or the internet access.’

### Theme IV: human resources and infrastructure

An environmental sanitation division usually consists of one district health officer, who has overall responsibility for dengue control, sprayer operatives, the number of which varies by district between 2 and 16, and one or two other staff, such as a security guard and a driver. Although only one staff member is required to operate a spraying machine, two machines are often in operation during a visit. A total of three staff, including a driver, may be required per site visit.

Sprayer operatives received annual training from the BMA Health Department. This training included spraying techniques, preparation of the insecticide chemicals, and self-protection in the event of a chemical spill. On the other hand, some officers complained about a lack of protective equipment and facilities, such as masks, protective overalls or even a shower room to wash themselves after the fumigation visit.

### Theme V: fumigation coverage area

Sprayer operatives aimed to spray inside a patient’s house, as well as around the outside of the house. Sometimes, house owners were reluctant to allow indoor spraying because they were worried about the safety of the spray, or they were worried about letting strangers into their house. For outdoor spraying, staff sometimes had problems accessing neighbours’ houses, either because they were not home or would not cooperate. Moreover, the chance of getting in to spray private residences, such as condominiums, townhouses, home offices, apartments and dormitories, was small due to the high level of security systems among these types of residences. Sprayers were allowed to spray indoors in less than 50% of residences.

Four new themes emerged from this study.

### New theme I: inefficient communication between hospitals, district offices and communities

Often information received from patients, e.g. their name, address or contact details, was inaccurate or not up-to-date. The district officers and the sprayer operatives thus had difficulty contacting the patient or a member of their household to arrange fumigation. The most common problem was that the contact number given did not match the house of the patient and staff had no other way to find the house. This mismatch of contact details could reflect genuine mistakes or it could be deliberate errors by households who did not wish their houses to be fumigated. In addition, the patient address given to hospital staff was not always the one where the patient was currently living.

One participant stated:

The patient’s contact details are sometimes wrong. We could not contact patients. Sometimes, patients give the details which are shown on their ID card, but these are not where they currently live.

In some cases of missing patient numbers, we try hard to find the patients. However, some we can find, and others we cannot find.

### New theme II: barriers to contact with patients via mobile phone using the office phone

The phone system used in all district offices was based on landlines, which would not allow staff to call out to mobile phones. However, with improving communication technology mobile phones have now become the main contact choice of people, including patients. There was no incentive for health staff to contact patients via their own personal mobile phone, since they would have had to absorb this cost themselves.

As one stated:

‘It is hard contacting patients using the landline, sometimes we use our own mobile phone, but we must support the cost by ourselves.’

### New theme III: benefits of community networks

This study suggested that community engagement in districts with low dengue incidence seemed to be much greater when compared with high incidence districts. This seemed to result from good relationships between district health officers and people in the communities, such as village headmen, assistant headmen, health volunteers and particularly residents themselves. The villagers often helped the health workers by preparing their residences for fumigation, for example, by moving bed-ridden patients, moving domestic animals and covering their property. In addition, these communities sometimes reported newly identified dengue cases in their community promptly by telephone, even before the health officer had received a notification through the reporting system. Such community participation would therefore appear to be an essential part of successful dengue control.

### New theme IV: concerns with fumigation chemicals

Chemical residuals remaining inside houses following fumigation, and inhalation of the chemicals were two main concerns, explaining why individuals were unwilling to allow health staff to spray inside their house.

As one staff member stated:

The householder said: It is not ok. The chemicals smell so bad! Do not spray in the house. How can we be sure there are no residual chemicals in our house?

The houses with some domestic animals were rejecting fumigation as they believed that their animals were sensitive to the chemical residuals and machine noises during the fumigation process.

One interviewee reported that:

‘The householder said: My bird is so expensive. Stop spraying now, if my bird died, who would be responsible?’

**Figure 3.**
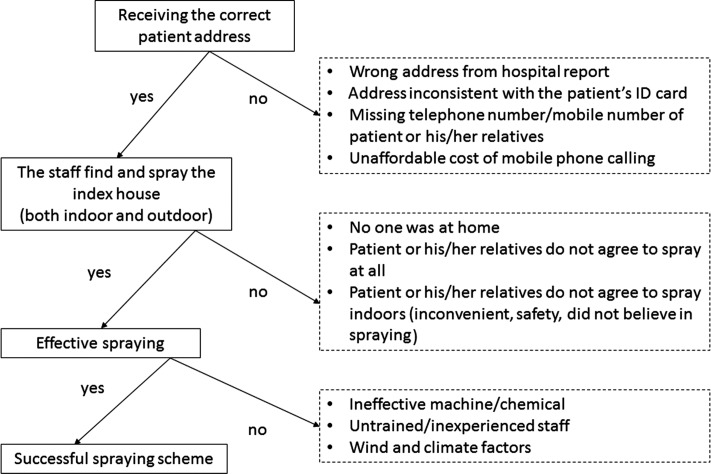
Summary of the spraying process and the challenges it faces.

Figure 3 summarizes the challenges that emerged during each step of the fumigation process to control dengue outbreaks. The problems faced by sprayer operatives and health staff could be grouped into three categories.

- The problem of insufficient or inaccurate patient contact details, which were necessary in order to identify addresses to be sprayed.
- The problems of accessing houses to spray due to various reasons, including a lack of community understanding and thus cooperation.
- Even if the first two categories were not an issue, ineffective resources including worn-out spraying machines, untrained staff, low quality chemical compounds and other external factors may still contribute to the unsuccessful fumigation process.

## Discussion

This study identified several problems with fumigation in the Bangkok setting. First, the failure to obtain complete and accurate patient information for all dengue cases, such as the patient’s phone number and address, could lead to delays in finding the house for spraying. In some cases, fumigation intervention could not be carried out at all. The second problem was a negative perception among some of the Bangkok population towards fumigation. Most interviewees stated that they had experience of householders refusing to allow them to spray inside their houses. Householders’ concerns include suspicion of the chemicals used for spraying, worries about harming their animals or other valuable items inside their houses, and reluctance to let strangers enter their house. However, some districts officers were intimately acquainted with the local communities, which facilitated their gaining permission to access houses for spraying.

The rationale for fumigation was to shut down an outbreak, which meant decreasing the infection rate and preventing the next generation of the outbreak. Any such response should be performed within 2 wk of the occurrence of the first case. Different strategies for fumigation have been shown to be effective worldwide.^18–20^ In contrast, the systematic and meta-analysis showed that indoor residual spray did not impact significantly on dengue infection risk. Moreover, insecticide aerosols were associated with higher dengue risk.^21^ As for Thailand, although there have been no recent reports of a successful fumigation campaign, one study mentioned an effective larval control programme in northeast Thailand,^22^ while others suggested that various control strategies, such as focal insecticide spraying^23^ and school-based control efforts,^24^ might be a good idea.

The need for complex resources and associated budgets, including machine maintenance, communication costs, personal protective equipment and training, was emphasized by the staff interviewed. All of these factors are required in order to mount effective control measures and should be carefully managed by local health offices.

A study of mosquito resting places revealed that more than 80% of mosquitoes circulated inside houses, for example, in bedrooms.^25^ This suggests that the optimum target location for fumigation was inside a patient’s house, in order to maximize the effectiveness of the control measures. However, in Bangkok, district health staff were often not allowed access to spray inside houses, especially patients’ houses. This was found to be the same in both high and low incidence areas.

In an urban setting, it is often more difficult to achieve community-wide collaboration. Many people were unavailable to prepare their houses for the spraying intervention to be performed, and were not available to let officers in to carry out fumigation. All district health officers interviewed stated that they actively encourage community participation in order to provide prompt and effective outbreak responses. Other studies that have conducted network analyses of dengue control in this region have also found that municipal governments play a key role in dengue control.^26–28^ These findings suggest that dengue control programmes could be made more effective through stronger collaborations with district offices.

## Conclusions

Thailand’s national dengue surveillance and control programme needs to improve communication between organizations, including hospitals, district offices and households. Community participation and social mobilization for behaviour modification were seen to be a good beginning towards improving the effectiveness of dengue control in an urban setting like Bangkok.

## Supplementary data

Supplementary data are available at *International Health* online (http://inthealth.oxfordjournals.org/).

## Supplementary Material

Supplementary Data
