# Supplementary material for: Addressing challenges faced by insecticide spraying for the control of dengue fever in Bangkok, Thailand: a qualitative approach
Source: Int Health. 2018 Jun 15;10(5):349–55. doi: 10.1093/inthealth/ihy038 (PMC6104709; doi:10.1093/inthealth/ihy038)
Supplement: Supplementary Data [file ihy038_supplementary_sprayingcontrolbkk_revised.docx]

**Supplementary 1**

*In-depth interview process*

This study adopted a qualitative approach by conducting face to face, in-depth interviews with ten designated district officers in their local BMA office. After the researcher had explained the study, the designated staff were given time to ask further questions before signing an informed consent document to join the study. The researcher did not receive any rejection to interview by the participants. The pre-determined themes were used while conducting the interviews. Approximately 45-minute interview took place in an office, field notes were made through the interview, and were tape recorded with the interviewees’ consent. The interview started with a general introduction as the researcher introduced oneself and briefly explained the background to the study, and assured the participants that any data collected would be strictly confidential. The participants were told to ask for clarification if any of the questions were not clear.

*Semi-structured interview*

The interviewees were asked to express their opinions about each pre-determined theme, including the length of their dengue control programme experience, challenges encountered during fumigation activities, and attitudes, perceptions, needs and expectations associated with their roles in the fumigation campaigns. Newly emerging themes were identified, while any deviations were uncoded during the analysis of the transcripts and the margin notes.

Open-ended questions

Pre-determined themes explored included the following:

*Theme I*: Standard practices regarding the MoPH and the BMA Health Department

1. What do the dengue surveillance and outbreak control plans involve?

*Theme II*: Machines - quality and quantity

1. What types of spraying machines are used and how many work effectively?

*Theme III*: Time to respond

a. Do they usually manage to do the spraying according to their timeline? If not, how long is the delay?

b. What do they do if there are some households where they cannot gain access the first time?

i. Do they go back and try to spray the house again?

ii. How much of a delay is there?

*Theme IV*: Human resources

a. How many employees work on the sprayer team for scheduled spraying, and how many for an outbreak situation?

b. Where and when did these workers receive their training?

*Theme V*: Fumigation coverage area

a. How many households can they gain access to spray, and have they reached their target number?

b. For each household, did they manage to do both indoor and outdoor spraying? If not, what was the problem?
